# A Study of Clinical Characteristics, Demographic Characteristics, and Fetomaternal Outcomes in Cases of Placenta Previa: An Experience of a Tertiary Care Center

**DOI:** 10.7759/cureus.32125

**Published:** 2022-12-02

**Authors:** Urmila Kumari, Ashok Naniwal, Vibha Rani, Ruchi Chandat, Seema Yadav, Dharmendra K Pipal

**Affiliations:** 1 Obstetrics and Gynaecology, Rabindranath Tagore Medical College, Udaipur, IND; 2 Obstetrics and Gynaecology, Pali Medical College, Pali, IND; 3 Gynaecology and Obstetrics, All India Institute of Medical Sciences, Gorakhpur, Gorakhpur, IND; 4 Obstetrics and Gynaecology, Satellite Hospital, Udaipur, IND; 5 Anaesthesia, Jaipur National University and Hospital, Jaipur, IND; 6 General, Colorectal and Minimal Access Surgery, All India Institute of Medical Sciences, Gorakhpur, Gorakhpur, IND

**Keywords:** cesarean section, postpartum hemorrhage, perinatal mortality, maternal mortality, preterm delivery, placenta previa

## Abstract

Background

This study aimed to determine the demographic and clinical characteristics of pregnant women presenting with placenta previa and study the risk factors for the development of placenta previa, management strategies of associated complications, and maternal and perinatal outcomes.

Methodology

This prospective, observational study was conducted in the Department of Obstetrics and Gynaecology at Dr. S.N. Medical College, Umaid Hospital, Jodhpur, Rajasthan, India from May to October 2019. All patients with placenta previa were studied based on clinical presentation, management, and fetal and maternal outcomes.

Results

A total of 10,041 patients delivered during the study period. Of these, 61 were diagnosed with either minor placenta previa (placental edge within 2 cm, not covering the internal os) or major placenta previa (placental edge reaching or overlapping the internal os) for an incidence of 0.6%. The majority of the cases (65.57%) were in the age group of 20-29 years. Among the cases of placenta previa, 13.11% had previous cesarean sections, and 9.83% underwent previous dilatation and curettage (D & C) procedures. Moreover, 78.68% of the cases had ultrasound findings of the placenta partially or completely covering the os. Most patients were delivered by cesarean section (96.7%), and only 3.27% were delivered by vaginal delivery. Intensive care unit admission was required in 14.75% of the cases. The most common maternal complications observed were antepartum and postpartum hemorrhage, transfusion of blood and blood products, and long hospital stays. The preterm delivery rate was 62.30%, and 37.70% were term deliveries. Almost half of the babies (49.18%) were born with a birth weight of ≥2.5 kg, and 50.81% were in the low-birth-weight category. Apgar scores >7 at five minutes were observed in 85.3% of cases. Neonatal intensive care unit (NICU) admissions were 39.34%, and most babies recovered and shifted to the mother’s side. The incidence of maternal mortality was 1.63%, and perinatal mortality was 9.83%.

Conclusions

The incidence of placenta previa was comparable to that reported in previous studies. Prevalence was more among younger women residing in rural areas who were unaware of regular antenatal check-ups. The main presenting symptom was painless vaginal bleeding, and ultrasonography was the most common diagnostic modality. Antepartum and postpartum hemorrhage was the most dreadful obstetric complications in cases of placenta previa, which affected maternal and fetal outcomes. Preterm and low birth weight were the main reason for NICU admissions. A team-based approach is required in the management of placenta previa cases.

## Introduction

Obstetric hemorrhage along with hypertension and infection are persistently posing threats to maternal health. It is the leading reason for the admission of pregnant women to intensive care units (ICUs) and contributes to almost all “near miss” cases in both developed and developing countries [1]. Obstetric hemorrhage contributes as a direct cause of maternal mortality in 25% of cases worldwide and 38% of cases in India [2].

Placenta previa is defined as the complete or partial covering of the placenta over the internal os of the cervix [3]. This condition comprises one-fifth of the cases of antepartum hemorrhage [4], as well as intrapartum and/or postpartum bleeding, with high risks for preterm birth and maternal, fetal, and neonatal morbidity. It occurs in 2.8/1,000 and 3.9/1,000 singleton and twin pregnancies, respectively [5]. Although the etiology of placenta previa is unknown, it is associated with damage to the uterine endometrium and scarring. It is correlated with advanced maternal age; multiparity; smoking; cocaine use; and previous history of suction and curettage, cesarean section, placenta previa, and assisted reproductive technology [3,6,7].

Regarding the distance of the placental margin to the internal os of the cervix, placenta previa occurs when the placental edge is within 2-3.5 cm of the internal os. Marginal or minor placenta previa occurs when the placental edge is within 2 cm of the internal os, and the term major or complete placenta previa is used when the placental edge reaches or overlaps the internal os [8]. This condition resolves by the time of delivery in 87.43% of women due to placental migration toward the upper uterine segment. The posterior placenta is found to have a higher rate of resolution at earlier gestation in the non-scarred uterus than the anterior placenta previa [9]. When the placental margin is 2 cm from the internal os or covering the os on a second-trimester ultrasound examination, a follow-up transvaginal ultrasound examination of the placental position should be performed at 32 and 36 weeks of gestation [10].

Vaginal bleeding not associated with pain during the second or third trimester of pregnancy is the characteristic presenting feature of placenta previa, and bimanual examination should be avoided as it may cause massive hemorrhage [5]. The placenta accreta spectrum should be considered in cases of placenta previa and should be ruled out at the earliest. The placenta accreta is the attachment of the placenta beyond the normal boundary of the myometrium that is established by the Nitabuch fibrinoid layer. Placenta increta is the invasion of the placenta into the myometrium, and placenta percreta is the invasion into the uterine serosa and/or the surrounding organs [11].

Elective cesarean section should be planned at 36 to 37 weeks in cases of uncomplicated placenta previa. Patients with severe antepartum hemorrhage should receive adequate volume replacement and packed red blood cell transfusion to maintain hemodynamic status, and termination of pregnancy should be done by emergency cesarean section, regardless of gestational age [12].

A systematic review of 10 studies on 600 patients with a low-lying placenta reported the outcomes of trials of labor by comparing the distance between the placental edge and the internal os. The results were as follows: (i) 0-10 mm distance - vaginal delivery: 43% (95% confidence interval (CI) = 28-59), emergency cesarean: 45% (95% CI = 22-69); (ii) 11-20 mm distance - 85% vaginal delivery (95% CI = 70-96), 14% emergency cesarean (95% CI = 4.2-29); and (iii) >20 mm distance - vaginal delivery: 82% (95% CI = 58-97), emergency cesarean: 10% (95% CI = 2.2-22.3) [13].

The present study aimed to determine the clinical and demographic profile of patients with placenta previa and evaluate the factors contributing to the complications, their management, and fetomaternal outcomes.

## Materials and methods

This is a prospective observational study carried out in the Department of Obstetrics and Gynaecology at Dr. S.N. Medical College, Umaid Hospital, Jodhpur, from May to October 2019. The study was approved by the Institutional Ethics Committee (SNMC/IEC/2019/92).

All patients with placenta previa diagnosed by ultrasonography after 28 weeks of pregnancy who were admitted to the hospital and followed till delivery were included in the study. Baseline demographic characteristics such as maternal age, parity, booked/unbooked, referral status, socioeconomic status, religion, and gestation age at the time of admission were noted. We excluded pregnant women with placenta previa who delivered outside our hospital.

All booked patients were counseled about the associated high risk and complications, the need for blood transfusion, operative delivery, and neonatal complications related to prematurity. Patients with complaints of bleeding were admitted, and the rest were treated on an outpatient basis. Antenatal corticosteroids were administered in cases of expected preterm delivery. Maternal and perinatal outcomes were recorded.

The maternal outcomes assessed included antepartum and postpartum hemorrhage, cesarean hysterectomy, the number of blood transfusions, placental abruption, disseminated intravascular coagulation (DIC), ICU admission, need for laparotomy, and mortality. The fetal outcomes included prematurity, birth weight, Apgar score, and stillbirth.

## Results

A total of 10,041 patients delivered during the study period. Of these, 61 cases were diagnosed as either minor (placental edge within 2 cm, not covering the internal os) or major placenta previa (placental edge reaching or overlapping the internal os) for an incidence of 0.6%. Most cases were unbooked, from rural populations, and belonged to lower socioeconomic status. The majority of these patients were Hindu. There were more referred patients than registered cases. These findings show that more such patients are referred to tertiary care centers from peripheral centers. The majority of women were in the 20-29-year age group and were nulliparous. The gestational age at the time of admission was 28 to ≥36 weeks. Painless vaginal bleeding was the most common presenting symptom (90.16%). One patient presented with hypovolemic shock. A majority of the patients (72.13%) came with ultrasonography, suggestive of placenta previa. Regarding classification, 78.68% of the cases were in the major placenta previa group, while 21.31% were in the minor (low-lying) placenta previa category (Table 1).

**Table 1:** Demographic and clinical features of patients with placenta previa. USG = ultrasonography; S/O = suggestive of; PP = placenta previa

Demographic features		Number of patients	Percentage
Booking status	Booked	20	32.7
	Unbooked	41	67.21
Residential status	Urban	16	26.22
	Rural	45	73.77
Socioeconomic status	Upper	6	9,83
	Middle	21	34.42
	Lower	34	55.73
Religion	Hindu	44	72.13
	Muslim	17	27.86
Referred	Yes	38	62.29
	No	23	37.70
Maternal age in years	<19	02	3.37
	20–29	40	65.57
	30–35	12	19.67
	>35	07	11.47
Parity	Nulliparous	38	62.38
	Multiparous	23	37.72
Gestation age at admission	28–33 weeks + 6 days	16	26.22
	34–36 weeks	22	36.06
	>36 weeks	23	37.71
Signs and symptoms	Painless vaginal bleeding	55	90.16
	Bleeding with pain abdomen	05	8.19
	Shock and hypotension	01	1.63
USG findings	S/O PP	44	72.13
	Not done	17	27.86
	Minor PP	13	21.31
	Major PP	48	78.68

**Figure 1:**
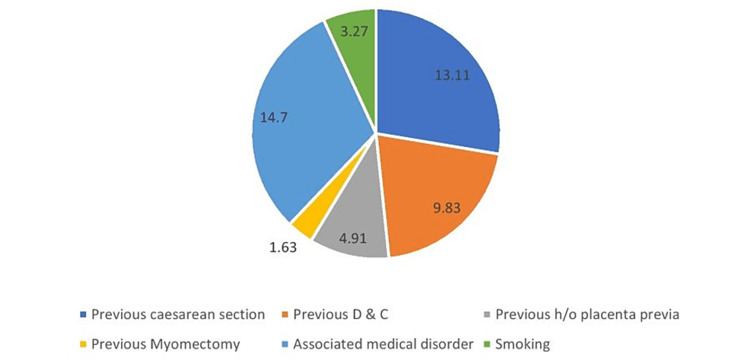
Risk factors among cases of placenta previa. D & C = dilatation and curettage; h/o = history of

In the placenta previa group, 13.11% of the cases had previous cesarean sections, and 9.83% had a history of previous dilatation and curettage procedures. A small proportion of the cases (14.7%) had an associated medical condition. Two cases had a history of smoking (Figure 1).

**Figure 2:**
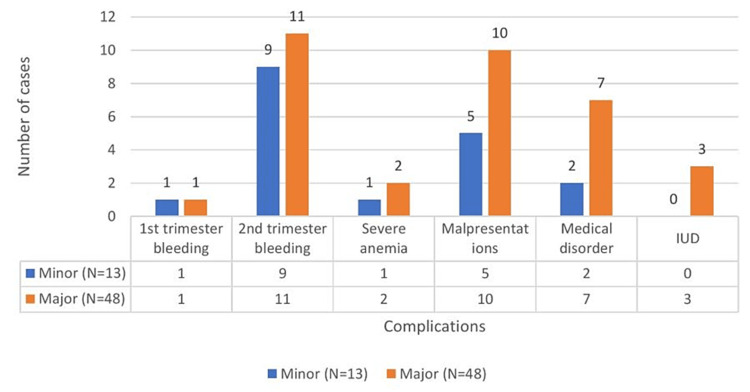
Antenatal complications. IUD = intrauterine device

In the antenatal period, 20 (32.78%) cases presented in the second trimester as antepartum hemorrhage and malpresentation were observed in placenta previa. Among these, there were three intrauterine deaths (Figure 2).

Regarding the management of placenta previa (Table 2), 93.44% of the cases required active management in the form of emergency cesarean sections at the time of admission, while four (6.55%) cases were managed expectantly till adequate lung maturity was achieved. Two of these cases were extremely preterm and another two were in early preterm gestation on admission. Overall, 73.77% of cases required emergency cesarean delivery, 26.22% of the patients underwent elective termination, and two (3.27%) cases were delivered by spontaneous vaginal delivery.

**Table 2:** Management of cases with placenta previa. CS = cesarean section; GA = gestational age

Management		Number of cases	Percentage
	Active	57	93.44%
	Expectant	04	06.55%
Mode of termination	Emergency CS	45	73.77%
	Elective CS	14	26.22%
	Spontaneous vaginal	02	03.27%
Expectant cases (n = 4)	GA on admission	GA on termination	Mode of termination
	32 weeks	34 weeks	Emergency CS
	24 weeks	36 weeks	Elective CS
	32.5 weeks	36 weeks	Emergency CS
	28 weeks	32 weeks	Emergency CS

**Figure 3:**
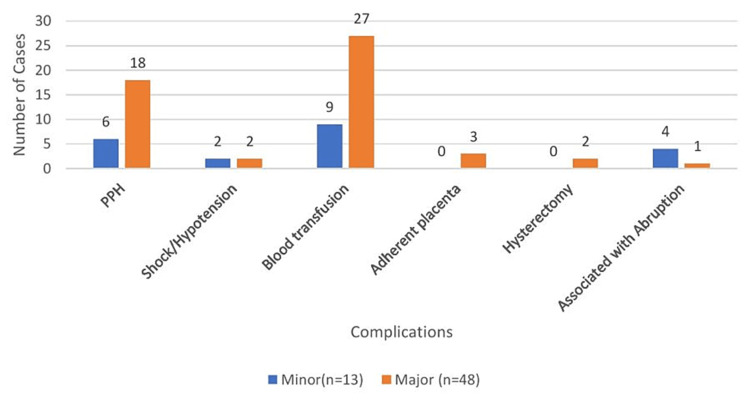
Intraoperative complications. PPH = postpartum hemorrhage

Figure 3 shows data on intraoperative complications, with postpartum hemorrhage being the most common (39.34%), and blood transfusion was done in 57.3% of cases. A small proportion of the cases (8.19%) also had a placental abruption, and 4.91% of cases were associated with the adherent placenta. Two patients required a cesarean hysterectomy.

**Figure 4:**
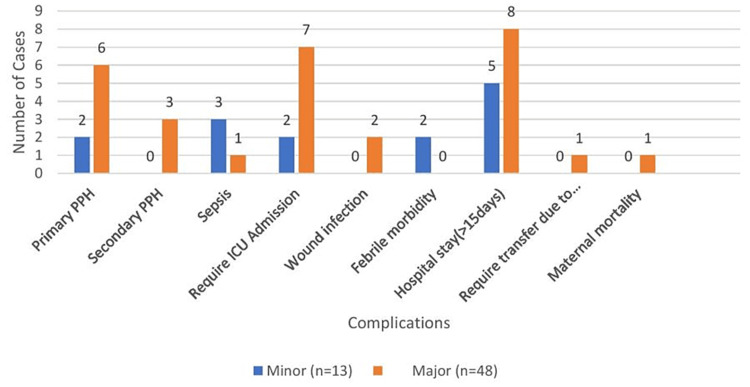
Postoperative complications. PPH = postpartum hemorrhage; ICU = intensive care unit

Figure 4 shows data on complications during the postoperative period in patients with placenta previa. Primary postpartum hemorrhage and secondary postpartum hemorrhage were the most common complications (13.11% and 4.91%, respectively). Hospital stay for >15 days and ICU admissions were 21.31% and 14.75% among major and minor groups, respectively. One case of maternal mortality was observed in the major placenta previa group.

Table 3 shows the different management strategies used in the cases of postpartum hemorrhage in our study. More than half of the cases (59%) required blood transfusion, 27.86% of the patients were managed by uterine balloon tamponade, and uterine artery ligation was done in 18.03% of the cases. Other procedures, such as B-Lynch sutures and internal iliac artery ligation, were conducted in 3.27% of the cases of postpartum hemorrhage. Non-pneumatic anti-shock garment (NASG) was applied in 6.55% of cases to prevent hypovolemic shock. A tiny proportion of the cases (3.27%) required a postpartum hysterectomy, and 6.55% of the patients were managed on vasopressors and ventilator support. This highlights that major placenta previa cases require more extensive surgical approaches in managing postpartum hemorrhage compared to cases with minor placenta previa. A total of 35 packed red blood cells were transfused, followed by 15 fresh frozen plasma, five platelet, and two cryoprecipitates.

**Table 3:** Management of PPH. PPH = postpartum hemorrhage; NASG = non-pneumatic anti-shock garment

Management	Minor (N = 13)	Major (N = 48)	Out of total 61 cases	Percentage
Uterine balloon tamponade	02	15	17	27.86
Uterine artery ligation	-	11	11	18.03
B-Lynch suture applied	-	01	01	3.27
NASG applied	02	02	04	6.55
Internal iliac artery ligation	-	02	02	3.27
Hysterectomy	-	02	02	3.27
Blood transfusion	09	27	36	59.01
Vasopressor support/mechanical ventilation used	01	03	04	6.55

Table 4 shows that a total of 34 neonates, out of 61 outcomes, shifted to the mother’s side. In total, 24 neonates were admitted to the NICU, of which 21 neonates recovered and were shifted to the mother’s side. Six perinatal deaths were recorded.

**Table 4:** Perinatal outcomes NICU = neonatal intensive care unit

Perinatal outcomes	Number of neonates	Percentage
Shifted to mother’s side	34	55.74
NICU admission	24	39.34
NICU recovered	21	34.42
Mortality	06	09.83

A total of five perinatal deaths were observed in the early preterm and one in late preterm cases. All newborns survived in the term gestation age group of patients (Table 5).

**Table 5:** Correlation between perinatal mortality and gestational age.

Gestational age (weeks)	Number of cases	Perinatal deaths	Percentage
28–33	16	05	31.25
34–36	22	01	4.55
37+	23	00	00
Total	61	06	9.84

Table 6 shows that out of six perinatal deaths, three were in the very low-birth-weight category (<1,500 g), and respiratory distress syndrome was the most prevalent cause apart from birth asphyxia and prematurity (Table 7).

**Table 6:** Correlation between perinatal mortality and birth weight.

Birth weight (in g)	Total number of birth	Perinatal mortality	Percentage
1,000–1,400	03	02	66.66
1,500–1,900	10	02	20
2,000–2,400	18	01	05.55
≥2,500	30	01	03.33

**Table 7:** Cause of death.

Cause of death	Number of cases	Percentage
Asphyxia	02	3.27
Prematurity	01	1.63
Respiratory distress syndrome	03	4.91

## Discussion

The incidence of placenta previa in this study was 0.6% (six per 1,000 deliveries). Previous studies have reported an incidence of placenta previa of 1.9% [14] and 0.2-4.84% [15,16]. A systematic review and meta-analysis showed high prevalence among Asian countries (12.2 per 1,000 pregnancies) and lower prevalence in Europe (3.6 per 1,000 pregnancies), North America (2.9 per 1,000 pregnancies), and Sub-Saharan Africa (2.7 per 1,000 pregnancies), though the true relationship between prevalence and ethnicity is yet to be established [17].

The majority (67.21%) of the cases were unbooked and from rural backgrounds (73.77%). This is due to the high burden of referred cases from peripheral health centers to the tertiary care center, which caters to the large population of western Rajasthan.

Over half of the cases (55.73%) were from the lower socioeconomic class, and only 9.85% were from the upper socioeconomic class. This can be explained by factors such as a lack of antenatal care awareness, a low literacy rate, poor medical facilities in the periphery, social customs, and a lack of control over predisposing factors such as an increased rate of cesarean sections and multiple abortions by untrained persons. More patients were from the Hindu (72.13%) religion compared to the Muslim (27.86%) because the majority of the population in the region is from the Hindu community.

Regarding age and parity, 65.57% of the cases were in the age group of 20-29 years, and the majority of the cases (62.38%) were nulliparous, while 37.62% were multiparous. Similar findings were observed in other studies as well, except that multiparity was more commonly found in placenta previa. This is contrary to our study, where nulliparous cases were more prevalent [18]. A minor proportion of the cases (13.11%) of placenta previa had previous cesarean sections, and 9.83% had previous dilatation and curettage procedures, which are considered important risk factors [14,19,20].

The gestation age at the time of admission was between 28 and 36 weeks, with the most common presentation being painless vaginal bleeding. A majority of the cases (72.13%) were diagnosed as placenta previa on ultrasonography findings, and the majority (78.68%) were partially or completely covering the os. Ultrasound has good accuracy in diagnosing this condition with 90% sensitivity and 98% specificity [21].

Most patients were delivered via cesarean section (96.7%), and only 3.27% were delivered via vaginal delivery. ICU admission was required in 14.75% of the cases, and ventilator support was required in 6.55%. The most common maternal complications were antepartum and postpartum hemorrhage, transfusion of blood and blood products, and long hospital stays. We observed one maternal death (1.63%) in our study. These findings were also observed in similar studies [16,22,23].

Regarding perinatal outcomes, the preterm delivery rate was 62.30%, and 37.70% were term deliveries. Almost half of the babies (49.18%) were born with a birth weight of ≥2.5 kg, and 50.81% were in the low-birth-weight category. In 85.3% of the cases, the Apgar score was >7 at five minutes of birth. NICU admissions were 39.34%, and most of the babies recovered and shifted to the mother’s side. The incidence of perinatal mortality was 9.83% caused by prematurity, birth asphyxia, and respiratory distress syndrome. Similar perinatal outcomes were reported in previous studies [21,23].

In this study, we included a large sample of participants as this is a tertiary care center that caters to a large population in the western part of Rajasthan state; however, the limitation is that this is a single-center study and multicenter data are needed to emphasize the role of interventional radiology such as embolization in the management of placenta previa cases.

## Conclusions

The prevalence of placenta previa in our study was 0.6% which was more prevalent among younger women residing in rural areas who were unaware of regular antenatal check-ups. The main presenting symptom was painless vaginal bleeding, and ultrasonography was the most common diagnostic modality. Antepartum and postpartum hemorrhage was the most dreadful obstetric complications in cases of placenta previa, which adversely affected maternal and fetal outcomes. Uterine procedures pose a risk of developing placenta previa, such as previous cesarean sections and dilatation and curettage procedures. Termination of pregnancy at earlier gestation and low birth weight were the reasons for NICU admissions. We observed 1.63% maternal mortality and 9.83% perinatal mortality.

This study highlights the importance of antenatal care. Adherence to antenatal clinic visit protocol, early diagnosis based on ultrasonography, institutional deliveries, and timely referral to a higher center with a competent team of skilled obstetricians, anesthetists, expert neonatologists, and blood bank facilities are needed to manage cases of placenta previa. Therefore, a comprehensive emergency obstetric care team well-trained in postpartum hemorrhage management and the availability of blood and blood products are key components in reducing maternal and perinatal morbidity and mortality in cases of placenta previa. A balanced approach should be adopted to lower the rate of primary cesarean sections and incorporate family planning counseling to reduce unnecessary uterine procedures.
